# Antagonism of Kinin Receptors B1 and B2 Attenuates Folic Acid‐Induced Tubulointerstitial Fibrosis in Mice

**DOI:** 10.1111/bcpt.70189

**Published:** 2026-01-20

**Authors:** Gabriel Rufino Estrela, Alexandre Budu, Juliene Silva, Raisa Brito, Frederick Wasinski, Jonatan Barrera‐Chimal, Ronaldo Carvalho Araujo

**Affiliations:** ^1^ Department of Biophysics Federal University of São Paulo São Paulo Brazil; ^2^ Discipline of Nephrology Federal University of São Paulo São Paulo Brazil; ^3^ Deparment of Neurology and Neuroscience Federal University of São Paulo São Paulo Brazil; ^4^ Maisonneuve‐Rosemont Hospital Research Centre Montreal Canada

**Keywords:** folic acid nephropathy, kinin receptors, tubulointerstitial fibrosis

## Abstract

Chronic kidney disease (CKD) remains a significant global health burden despite recent advances in pharmacotherapy, including sodium‐glucose cotransporter 2 (SGLT2) inhibitors and mineralocorticoid receptor antagonists. Renal fibrosis, a central pathological hallmark of CKD progression, is mediated by persistent inflammation and macrophage activation, wherein the kallikrein–kinin system—particularly kinin receptors—plays a critical role. Emerging evidence supports the therapeutic potential of dual kinin receptor antagonism, especially targeting B1R, though the precise molecular mechanisms remain incompletely understood, necessitating further investigation. To elucidate the role of kinin receptors in renal injury, male C57BL/6 mice were subjected to folic acid‐induced nephropathy and treated with either the B1R antagonist R715 or the B2R antagonist HOE140. Treatments were administered pre‐ and postfolic acid injection. Renal function was evaluated via serum creatinine, blood urea nitrogen and urine analyses. Renal tissues underwent histopathological assessment and gene expression profiling to assess injury and fibrotic responses. B2R antagonism (HOE140) failed to attenuate acute tubular injury but ameliorated chronic damage by downregulating proinflammatory mediators and upregulating anti‐inflammatory markers. In contrast, B1R blockade (R715) exacerbated acute kidney injury yet mitigated chronic fibrosis, improving renal function and reducing profibrotic gene expression. These findings delineate distinct, time‐dependent roles of B1R and B2R in modulating macrophage polarization (M1/M2) and fibrogenesis, identifying potential targets for antifibrotic therapies.

## Plain Language Summary

Chronic kidney disease affects millions of people and often worsens because of long‐term inflammation and scarring in the kidneys. This study used mice to test how two inflammation‐related proteins, called kinin B1 and B2 receptors, influence kidney damage. Blocking the B2 receptor did not help early kidney injury but reduced long‐term inflammation. Blocking the B1 receptor made early injury worse but later improved kidney function and reduced scarring. These findings show that the two receptors play different roles over time and may help guide future treatments.

## 1 Introduction

Chronic kidney disease (CKD) is a worldwide public health issue with a high index of mortality and that despite the newly introduced therapeutic agents to slow the progression of CKD to end‐stage renal disease (ESRD), including SGLT2 inhibitors and nonsteroidal mineralocorticoid receptor antagonists [1, 2, 3, 4, 5, 6, 7], there is a residual risk that needs to be targeted and therefore the need to identify novel therapeutic options. Fibrosis is the main pathological process in CKD progression, regardless of the cause of initial renal injury. Renal fibrosis is characterized by extracellular matrix accumulation, myofibroblast activation and inflammatory responses that lead to renal dysfunction. Infiltration of inflammatory cells early after acute kidney injury (AKI) episode has an important role to predict an effective or a maladaptive repair [8]. Recruitment and activation of macrophages is the most important factor behind fibrotic diseases. Macrophages adopt different phenotypes, such as M1 proinflammatory cells, which can promote renal injury, and M2 anti‐inflammatory cells, which have a reparative phenotype and contribute to resolution phase of inflammatory process [9, 10]. Kinins are well known to affect the inflammatory response by regulating leukocyte recruitment, neutrophil and macrophage activation, release of prostaglandins, cytokines and chemokines [11, 12, 13, 14, 15, 16, 17]. Their action is mediated by two G‐protein coupled transmembrane receptors, kinin B2 receptor (B2R), which is responsible for most kinins effects and is constitutively expressed, and kinin B1 receptor (B1R), which presents lower expression levels and presents upregulation after inflammatory stimuli [18, 19]. We observed that both kinins receptors antagonism and deletion have been shown to attenuate cisplatin nephrotoxicity [20, 21]; their role was also observed in several other models of kidney disease, such as renal ischemia–reperfusion, which have demonstrated that both B1R antagonism and genetic deletion can mitigate the transition from AKI to CKD by promoting an increase in the M2 macrophage population. This finding corroborates an earlier study indicating reduced inflammation in B1R knockout mice following renal ischemia–reperfusion. Furthermore, early activation of the B2R has been shown to exacerbate renal ischemia–reperfusion injury. Additionally, B1R blockade has been found to be beneficial in conditions such as glomerulosclerosis, obstructive nephropathy and renal injury induced by hypertension in rodent models [22, 23, 24, 25, 26]. Recently, our group showed that kinin B1 receptor antagonism exacerbates cisplatin‐induced renal fibrosis, and that was due to less polarization of M2 macrophages to the injured tissue [27]. While cisplatin and ischemia–reperfusion (IR) models mainly focus on acute tubular necrosis and direct cytotoxic or ischemic injury mechanisms, the folic acid (FA) nephropathy model uniquely captures progressive tubulointerstitial fibrosis and chronic inflammation central to AKI‐to‐CKD transition. FA nephropathy allows detailed study of macrophage polarization dynamics and the roles of both kinin receptors in a more fibrotic, inflammation‐driven microenvironment that better mimics human CKD progression [28, 29]. This can provide us new insight into immune regulation and fibrosis resolution beyond the primarily acute injury contexts of cisplatin and IR models, complementing and extending prior findings in these models. Here, we want to investigate the effects of B1R and B2R antagonisms in folic acid nephropathy severity, in the acute phase and in CKD progression following the acute stimulus.

## 2 Methods

### 2.1 Animals

Male C57BL/6 mice, weighing between 23 and 28 g and aged 8 to 12 weeks, were used in the present experiments. The animals were obtained from the Animal Care Facility of the Federal University of São Paulo (UNIFESP). Mice were housed in groups of five per cage under standard laboratory conditions, with controlled temperature (22°C ± 2°C), relative humidity (50%–60%) and a 12‐h light/dark cycle. Animals had ad libitum access to standard rodent chow and filtered water. Environmental enrichment, such as nesting material, was provided in all cages. Animal welfare was closely monitored throughout the study. Following folic acid administration, mice were examined daily for signs of pain or discomfort using the Mouse Grimace Scale (MGS). Facial action units indicative of pain were systematically assessed. Humane end points were predefined and included sustained high MGS scores indicative of moderate to severe pain, body weight loss exceeding 15%–20% of baseline, marked reduction in mobility, grooming or signs of dehydration or hunched posture and any severe or persistent clinical signs of distress. Animals reaching any of these criteria were humanely euthanized to minimize suffering. All procedures underwent review and approval by the Institutional Animal Care and Use Committee (CEUA) of the Federal University of São Paulo, in compliance with the regulations set forth by the National Council for the Control of Animal Experimentation (CONCEA). The study was approved on 24 August 2019, under protocol number CEUA 3456260419. The study was conducted in accordance with the Basic & Clinical Pharmacology & Toxicology policy for experimental and clinical studies [30].

### 2.2 Experimental Protocol

Mice were randomly assigned by a trained investigator to one of the following experimental groups: vehicle, folic acid (FA), folic acid plus the B1 receptor antagonist R715 (FA + R715), or folic acid plus the B2 receptor antagonist HOE140 (FA + HOE140). Randomization was also applied during sample processing to minimize experimental bias. For each condition, 5–6 animals were used per group.

### 2.3 Folic Acid Nephropathy

A single intraperitoneal injection of folic acid (200 mg/kg; Sigma‐Aldrich, St. Louis, MO, USA) dissolved in a 0.3‐M NaHCO3 vehicle, or vehicle alone, was administered. Mice were euthanized 48 h for AKI and 28 days postinjection of folic acid for CKD.

### 2.4 Antagonist Treatment

Animals were administered 800 μg/kg i.p of either HOE140 (Sigma‐Aldrich, St. Louis, MO, USA) or R715 (Sigma‐Aldrich, St. Louis, MO, USA) at 48, 24 and 1 h prior to the folic acid injection, as well as 24 h following the treatment [20, 21, 23, 27, 31, 32].

### 2.5 Blood Sampling and Tissue Collection

The mice were anaesthetized via intraperitoneal injection of ketamine (91 mg/kg) and xylazine (9.1 mg/kg), and blood was subsequently collected through cardiac puncture. For serum preparation, blood samples were allowed to clot at room temperature for 2 h, followed by centrifugation at 2000 g for 20 min. The resulting serum was stored at −20°C. Kidney tissue was harvested, and the renal capsule was carefully removed. Transverse cuts were made, and the kidneys were immediately frozen in liquid nitrogen before being stored at −80°C.

### 2.6 Renal Function

Serum creatinine and urea levels were measured to assess renal function. Samples were analysed using commercially available colorimetric assay kits (Labtest, Lagoa Santa, Brazil). Urine was collected over a 24‐h period in metabolic cages 3 days prior to euthanasia, and protein concentration was determined using the Sensiprot assay kit (Labtest, Lagoa Santa, Brazil).

### 2.7 Real‐Time PCR

Kidney samples were immediately frozen at −80°C upon collection. Total RNA was extracted using TRIzol Reagent (Invitrogen, Carlsbad, CA, USA), and RNA integrity was verified through electrophoresis on an agarose gel. cDNA synthesis was performed using the High‐Capacity cDNA Reverse Transcription Kit (Applied Biosystems, Waltham, MA, USA). Standard curves were generated to assess the amplification efficiency of each primer pair. Real‐time PCR was conducted using the SYBR Green system (Thermo Scientific, Waltham, MA, USA) with primers specific to the target sequences. Primers were designed using Primer3 Web, and their specificity was confirmed using NCBI Primer‐BLAST, followed by synthesis (Exxtend, Campinas, Brazil). Target mRNA expression was normalized to 18S rRNA for SYBR assays and to Gapdh for TaqMan assays, and relative expression levels were calculated using the comparative threshold cycle (2^−ΔΔCt) method. All primer sequences are listed in the 5′–3′ orientation and presented in Table 1. TaqMan probe assays included Bdkb1r (Mm00432059_s1), Bdkb2r (Mm00437788_s1) and Gapdh (Mm99999915_g1).

**TABLE 1:** Sequences of primers used in real‐time PCR assays.

Gene	Forward primer (5′–3′)	Reverse primer (5′–3′)
*Rps18*	CGC CGC TAG AGG TGA AAT TC	TCT TGG CAA ATG CTT TCG C
*Actb*	CTG GCC TCA CTG TCC ACC TT	CGG ACT CAT CGT ACT CCT GCT T
*Lcn2*	ATG TGC AAG TGG CCA CCA CG	CGC ATC CCA GTC AGC CAC AC
*Tnf*	GCC TCT TCT CAT TCC TGC TTG	CTG ATG AGA GGG AGG CCA TT
*Havcr1*	TGT CGA GTG GAG ATT CCT GGA TGG T	GGT CTT CCT GTA GCT GTG GGC C
*Tgfb1*	CAA CAA TTC CTG GCG TTA CCT TGG	GAA AGC CCT GTA TTC CGT CTC CTT
*Col3a1*	TGG ACC AAA AGG TGA TGC T	CAA GAC CTC GTG CTC CAG T
*Col4a1*	TCC CTG GTA GTC GTG GAG A	GCC TGC TTG TCC TTT TTC A
*Vim*	CAG GAG GAG ATG CTC CAG A	AGG TCA AGA CGT GCC AGA G
*Arg1*	CGC CTT TCT CAA AAG GAC AG	CCA GCT CTT CAT TGG CTT TC
*Mrc1*	CAA GGA AGG TTG GCA TTT GT	CCT TTC AGT CCT TTG CAA GC
*Il4ra*	CAC AGT GCA CGA AAG CTG AA	ATG GGC ACA AGC TGT GGT AG
*Chi3l3*	CCC CTG GAC ATG GAT GAC TT	AGC TCC TCT CAA TAA GGG CC
*Col1a1*	CCC CGG GAC TCC TGG ACT T	GCT CCG ACA CGC CCT CTC TC
*Acta2*	TTG GAA AAG ATC TGG CAC CAC	GCA GTA GTC ACG AAG GAA TAG
*Fn1*	CCT ACG GCC ACT GTG TCA CC	AGT CTG GGT CAC GGC TGT CT
*Il1b*	AGG AGA ACC AAG CAA CGA CA	CGT TTT TCC ATC TTC TTC TTT G
*Il6*	TAG TCC TTC CTA CCC CAA TTT CC	TTG GTC CTT AGC CAC TCC TCC
*Cd86*	TCA AGG ACA TGG GCT CGT ATG	AGG TTC ACT GAA GTT CGC GAT

*Note: Rps18* (18S ribosomal RNA), *Actb* (beta‐actin), *Lcn2* (lipocalin 2, also known as NGAL), *Tnf* (tumour necrosis factor alpha, TNF‐*α*), *Havcr1* (kidney injury molecule‐1, KIM‐1), *Tgfb1* (transforming growth factor beta 1, TGF‐*β*1), *Col3a1* (collagen type III alpha 1), *Col4a1* (collagen type IV alpha 1), *Vim* (vimentin), *Arg1* (arginase 1), *Mrc1* (mannose receptor C‐type 1, CD206), *Il4ra* (interleukin‐4 receptor alpha, IL‐4R), *Chi3l3* (chitinase‐like protein 3, Ym1), *Col1a1* (collagen type I alpha 1), *Acta2* (alpha‐smooth muscle actin, *α*‐SMA), *Fn1* (fibronectin 1), *Il1b* (interleukin‐1 beta, IL‐1*β*), *Il6* (interleukin‐6) and *Cd86* (cluster of differentiation 86).

### 2.8 Renal Histopathology Analysis

The kidneys were fixed in 10% formaldehyde, followed by dehydration and embedding in paraffin. Sections (4 μm) were cut and stained with haematoxylin–eosin and Sirius red. At least six subcortical fields per mouse were examined and analysed using a Leica DM4000 microscope at 200× magnification. Tubular injury scores were assigned based on the percentage of tubules exhibiting luminal casts, cell detachment or dilation, according to the following scale: 0 = 0 to 5%, 1 = 6 to 25%, 2 = 26 to 50%, 3 = 51 to 75% and 4 = > 75%. Histological analysis was performed in a blinded manner to the experimental groups to assess tubule‐interstitial fibrosis, based on the area positively stained with picrosirius red, using the following scale: 1 = 25%, 2 = 26 to 50%, 3 = 51 to 75% and 4 = > 75%.

### 2.9 Statistical Analysis

All data are expressed as the mean ± standard error of the mean (SEM). Continuous variables were analysed using one‐way analysis of variance (ANOVA) followed by Tukey's post hoc test for multiple comparisons. Ordinal data from histological assessments were analysed using the Kruskal–Wallis test followed by Dunn's post hoc test. Statistical significance was set at *p* < 0.05. All statistical analyses were performed using GraphPad Prism 10 (GraphPad Software, La Jolla, CA, USA).

## 3 Results

### 3.1 Kinin B2R Antagonism Did Not Attenuate Folic Acid‐Induced Acute Tubular Injury

**FIGURE 1:**
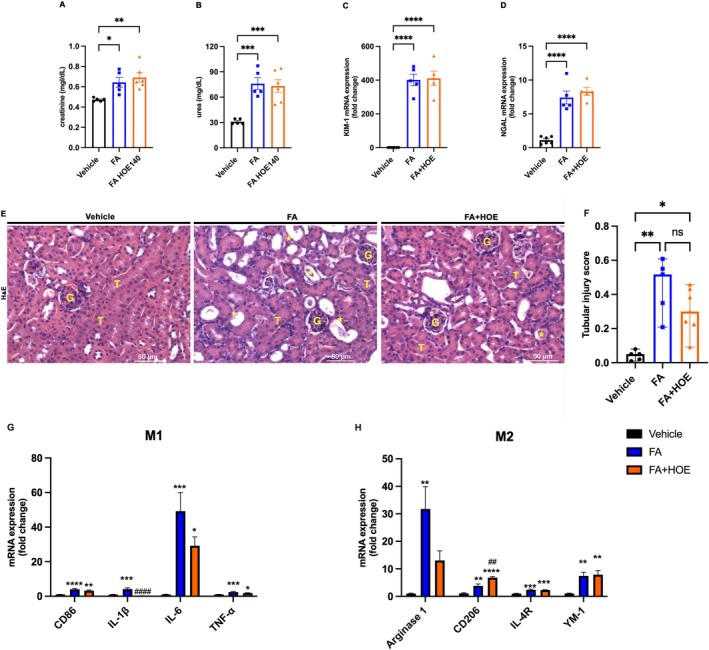
Kinin B2 receptor antagonism did not prevent renal dysfunction or tubular injury in a model of folic acid‐induced acute kidney injury. Renal function was assessed 48 h after folic acid injection by measuring serum creatinine (A) and urea (B) levels. Kidney injury markers KIM‐1 (C) and NGAL (D) were analysed by quantitative real‐time PCR. Tubular injury scores were obtained from haematoxylin and eosin–stained kidney sections (E–F). M1 and M2 macrophage markers were evaluated by real‐time PCR (G–H) across three experimental groups: Vehicle, FA (folic acid–treated) and FA + HOE140 (folic acid treated with the B₂ receptor antagonist HOE140). Continuous data (creatinine, urea and gene expression levels) are presented as the mean ± SEM and were analysed using one‐way ANOVA followed by Tukey's post hoc test. Ordinal data (tubular injury scores) are presented as medians with ranges and were analysed using the Kruskal–Wallis test followed by Dunn's post hoc test. Scale bars = 50 μm. *p < 0.05, **p < 0.01, ***p < 0.001 and ****p < 0.0001. *n* = 5–6 animals per group.

Renal function was assessed 48 h following folic acid injection. Creatinine and urea levels were elevated in both the FA and FA + HOE140 groups (Figure 1A–B). Additionally, treatment with HOE140 did not reduce the mRNA levels of KIM‐1 or NGAL (Figure 1C–D), and tubular injury was not attenuated in the HOE140‐treated group (Figure 1E–F). Next, we evaluated the mRNA levels of several proinflammatory and anti‐inflammatory markers and mediators. Kinin B2R antagonism reduced IL‐1*β* mRNA levels, but no significant differences were observed in the mRNA levels of other proinflammatory cytokines between the FA and FA + HOE140 groups (Figure 1G). Conversely, HOE140 treatment led to an increase in the anti‐inflammatory marker CD206 (Figure 1H).

### 3.2 Kinin B2R Antagonism Prevents the Development of Chronic Folic Acid Nephropathy

**FIGURE 2:**
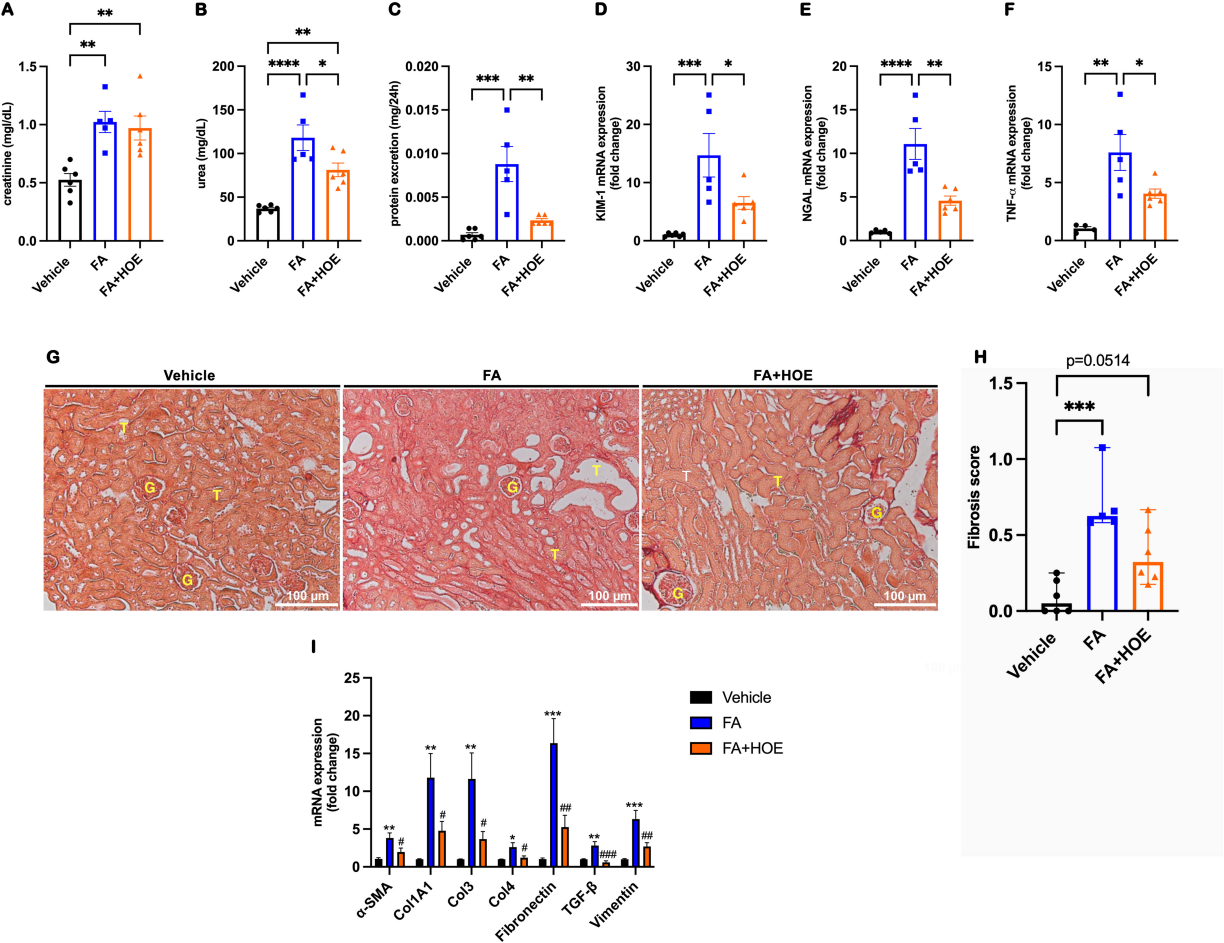
Kinin B2R antagonism alleviates renal dysfunction and reduces tubulointerstitial fibrosis induced by folic acid. Renal function was evaluated 28 days after folic acid injection by measuring serum creatinine (A), urea (B) and proteinuria (C). Kidney injury markers KIM‐1 (D), NGAL (E) and TNF‐*α* (F) were quantified by quantitative real‐time PCR. Renal fibrosis was assessed using picrosirius red‐stained kidney sections (G–H), and fibrotic marker expression was analysed in renal tissue by real‐time PCR (I) across three experimental groups: Vehicle, FA (folic acid–treated) and FA + HOE140 (folic acid treated with the B₂ receptor antagonist HOE140). Continuous data (creatinine, urea, proteinuria, and gene expression) are expressed as mean ± SEM and were analysed using one‐way ANOVA followed by Tukey's post hoc test. Ordinal data (fibrosis scores) are presented as medians with ranges and were analysed using the Kruskal–Wallis test followed by Dunn's post hoc test. Scale bars = 100 μm. *p < 0.05, **p < 0.01, ***p < 0.001 and ****p < 0.0001. *n* = 5–6 animals per group.

Kidney function was assessed 28 days following folic acid treatment. No significant differences in creatinine levels were observed between the FA and FA + HOE140 groups (Figure 1A). However, urea and protein excretion levels were significantly reduced in the HOE140‐treated group (Figure 2B,C). Additionally, renal mRNA levels of KIM‐1, NGAL and TNF‐*α* were significantly attenuated with HOE140 treatment (Figure 2D–F). Histopathological analysis of slides stained with picrosirius red revealed a reduction in collagen‐positive areas in the HOE140‐treated group (Figure 2G,H), a finding that was further supported by the decreased renal mRNA expression of several fibrotic markers (Figure 2I).

### 3.3 Kinin B1R Antagonism Exacerbates Folic Acid‐Induced AKI

**FIGURE 3:**
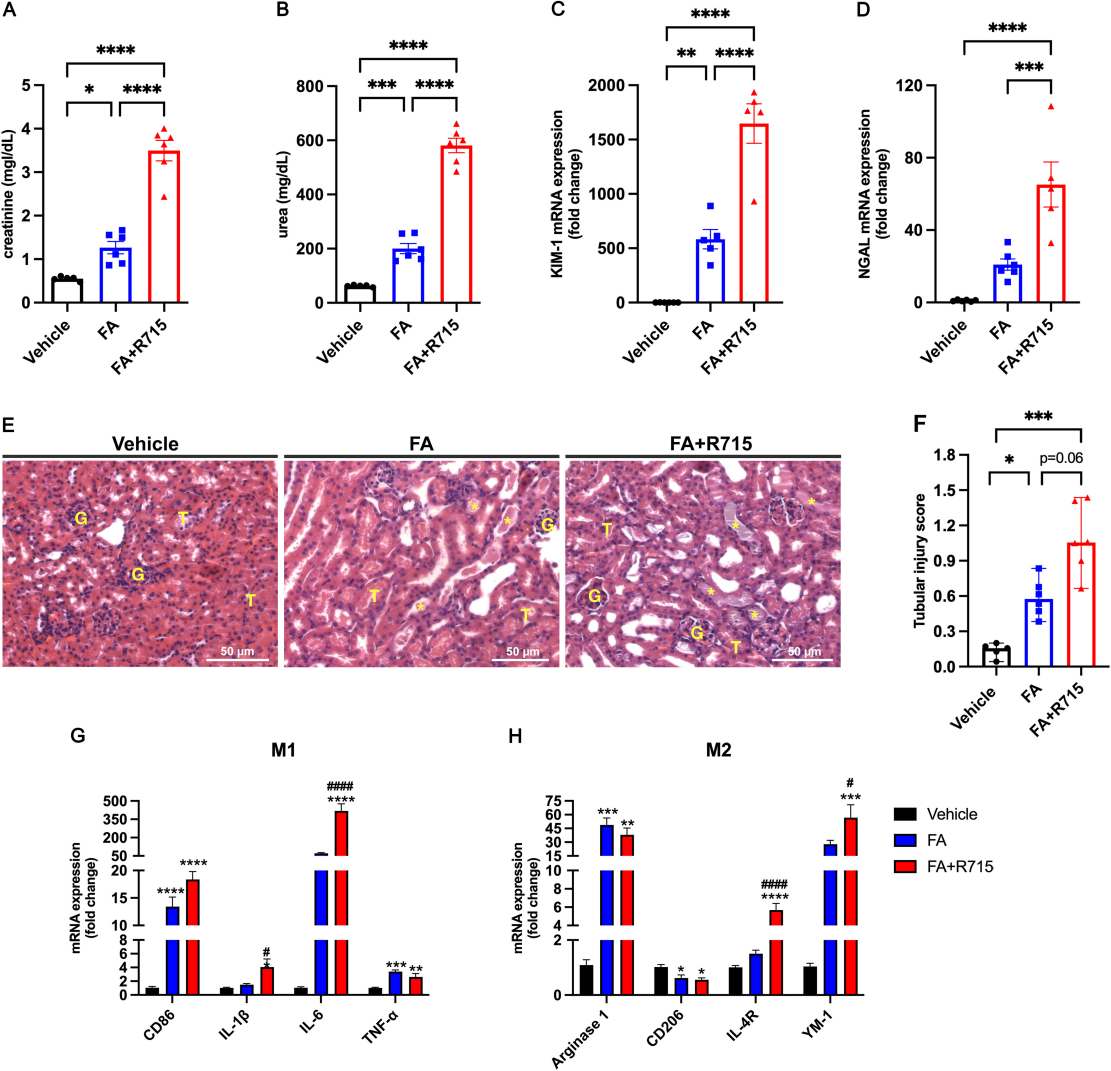
Kinin B1R antagonism worsens acute kidney injury induced by folic acid. Kidney function was assessed 48 h after folic acid administration by measuring serum creatinine (A) and urea (B) levels. Kidney injury markers KIM‐1 (C) and NGAL (D) were quantified by quantitative real‐time PCR. Tubular injury scores were determined from haematoxylin and eosin–stained kidney sections (E–F). M1 and M2 macrophage markers were analysed by real‐time PCR (G–H) across three experimental groups: Vehicle, FA (folic acid–treated) and FA + R715 (folic acid treated with the B₁ receptor antagonist R715). Continuous data (creatinine, urea and gene expression) are presented as mean ± SEM and were analysed using one‐way ANOVA followed by Tukey's post hoc test. Ordinal data (tubular injury scores) are presented as medians with ranges and were analysed using the Kruskal–Wallis test followed by Dunn's post hoc test. Scale bars = 50 μm. *p < 0.05, **p < 0.01, ***p < 0.001 and ****p < 0.0001. *n* = 5–6 animals per group.

Creatinine and urea levels were assessed 48 h following folic acid injection. Treatment with the Kinin B1R antagonist, R715, significantly elevated these parameters (Figure 3A,B). Furthermore, R715 treatment also exacerbated tubular injury following FA injection as shown by the increased mRNA levels of KIM‐1 and NGAL (Figure 3C,D), and that was corroborated by histopathological analysis of H&E‐stained slides, revealing increased tubular injury in R715‐treated mice (Figure 3E,F). Real‐time PCR analysis of kidney tissue showed an elevation in the mRNA levels of the proinflammatory cytokines IL‐1*β* and IL‐6 in the mice receiving the B1R antagonist as compared to FA alone (Figure 3G). Interestingly, R715 treatment also induced an increase in the mRNA expression of anti‐inflammatory markers IL‐4R and YM‐1 (Figure 3H).

### 3.4 Kinin B1R Antagonism Prevents the Development of Chronic Folic Acid Nephropathy

**FIGURE 4:**
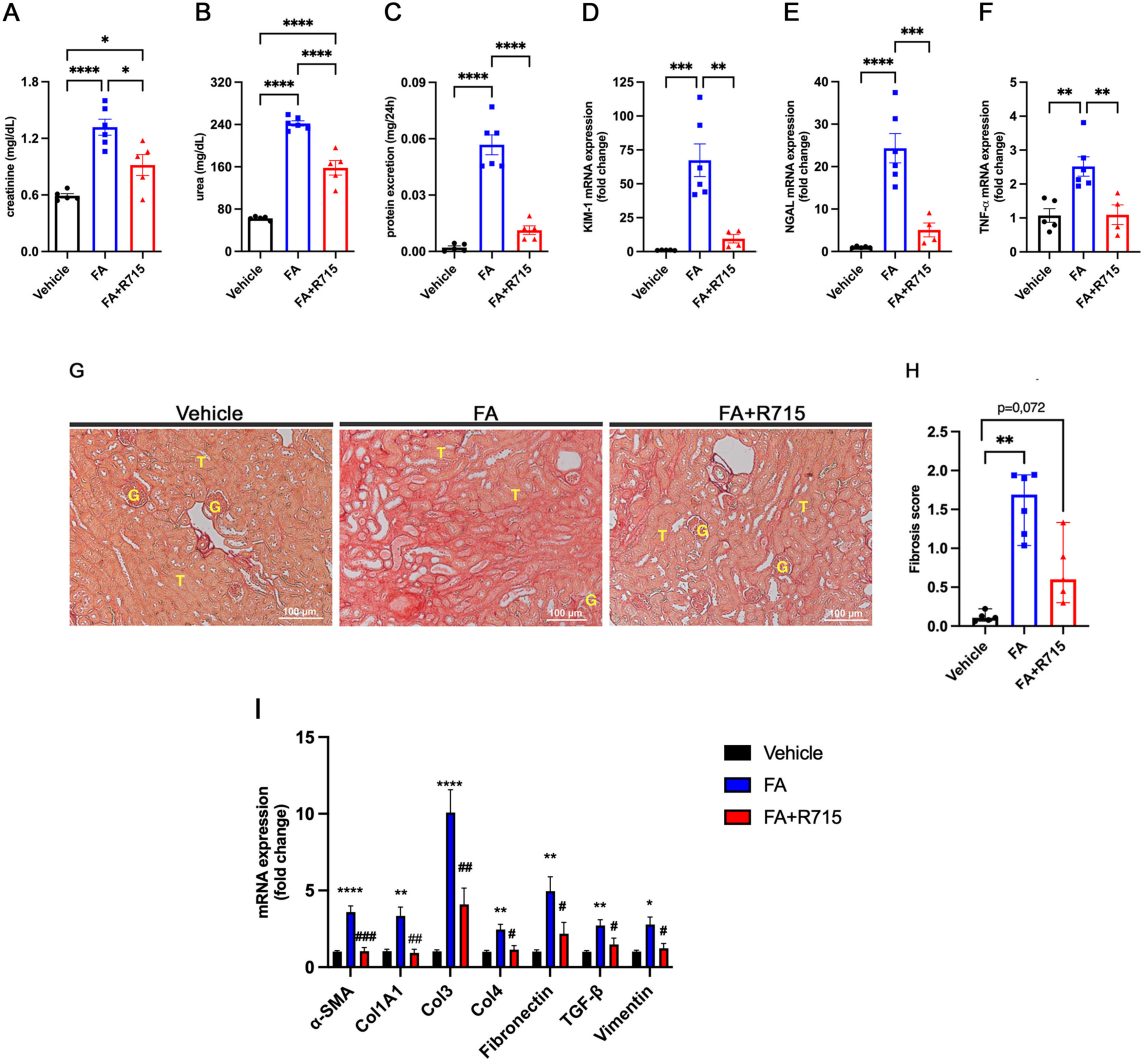
Kinin B1R antagonism prevents the progression of chronic kidney disease induced by folic acid. Renal function was evaluated 28 days after folic acid administration by measuring serum creatinine (A), urea (B) and proteinuria (C) levels. Kidney injury markers KIM‐1 (D), NGAL (E) and TNF‐*α* (F) were quantified by quantitative real‐time PCR. Renal fibrosis was assessed using picrosirius red‐stained kidney sections (G–H), and fibrotic marker expression was evaluated in renal tissue by real‐time PCR (I) across three experimental groups: Vehicle, FA (folic acid–treated) and FA + R715 (folic acid treated with the B₁ receptor antagonist R715). Continuous data (creatinine, urea, proteinuria and gene expression) are presented as mean ± SEM and were analysed using one‐way ANOVA followed by Tukey's post hoc test. Ordinal data (fibrosis scores) are presented as medians with ranges and were analysed using the Kruskal–Wallis test followed by Dunn's post hoc test. Scale bars = 100 μm. *p < 0.05, **p < 0.01, ***p < 0.001 and ****p < 0.0001. *n* = 4–6 animals per group.

Renal function was assessed 28 days following folic acid injections. Creatinine, urea and protein excretion levels were significantly reduced in animals treated with R715 when compared with the FA group (Figure 4A–C). Additionally, R715 treatment led to a significant reduction in the renal mRNA expression of KIM‐1, NGAL and TNF‐*α* to the FA group (Figure 4D–F). Histological analysis of picrosirius red‐stained slides revealed a reduction in collagen‐positive areas in the R715‐treated group compared to FA group (Figure 4G–H), a finding further supported by the decreased renal mRNA levels of fibrotic markers (Figure 4I).

### 3.5 Kinetics of Kinin Receptors and Macrophage‐Associated Gene Expression Over 28 Days

**FIGURE 5:**
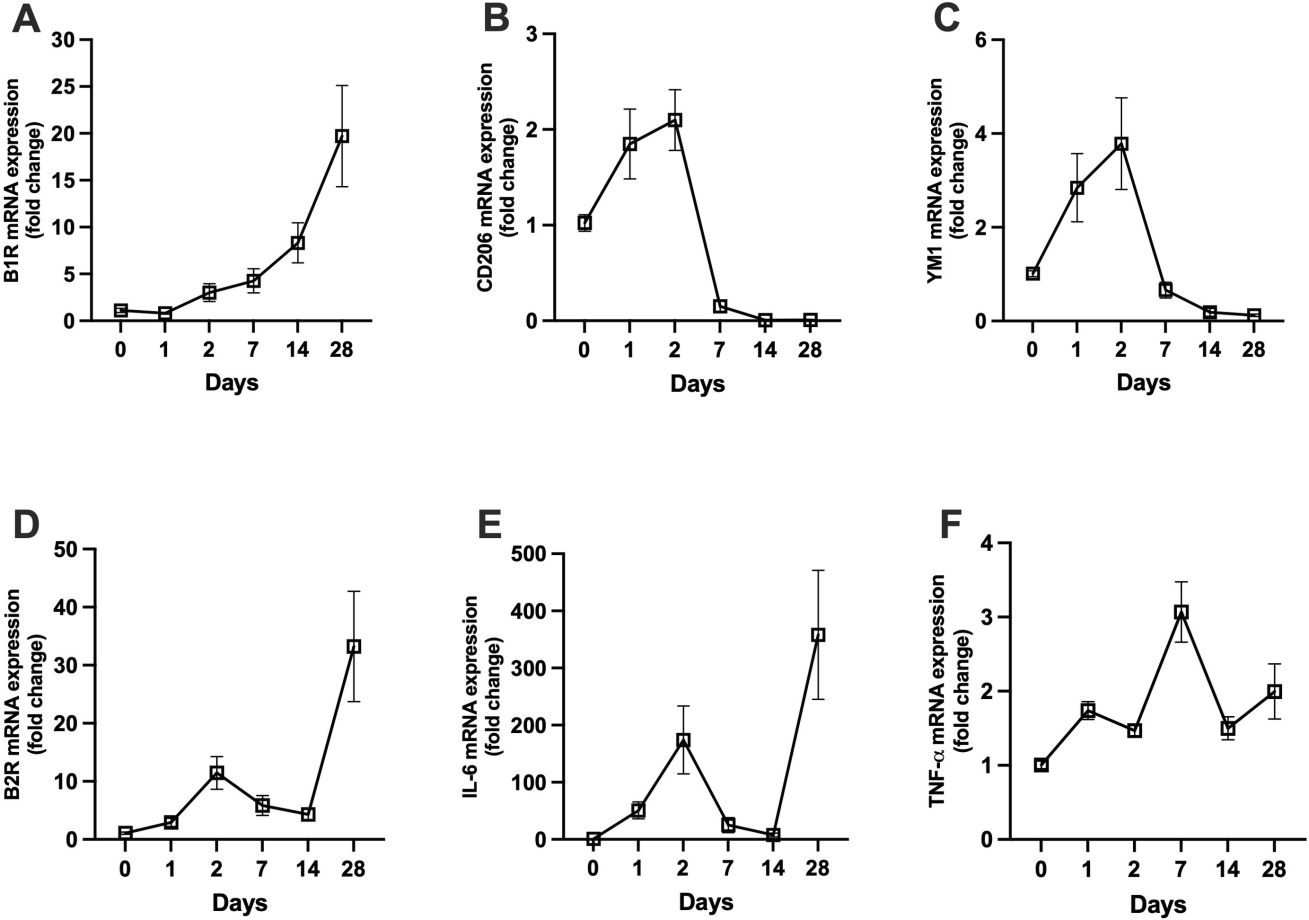
Temporal changes in renal B₁ and B₂ kinin receptor and M1/M2 macrophage marker mRNA expression following folic acid administration. Mice received a single intraperitoneal injection of folic acid (200 mg/kg), and renal tissues were collected at 0, 1, 2, 7, 14 and 28 days postinjection. Relative mRNA expression levels of kinin B₁ receptor (**A**), CD206 (**B**) and YM1 (**C**), as well as kinin B₂ receptor (**D**), IL‐6 (**E**) and TNF‐*α* (**F**), were quantified by quantitative real‐time PCR (qPCR). Data are presented as individual data points connected by lines to illustrate temporal changes. Each time point represents *n* = 5–6 animals.

To investigate the temporal regulation of kinin receptors, macrophage polarization markers mRNA expression was assessed over a 28‐day period. B1R mRNA expression increased progressively over time, peaking at day 28 (~20‐fold) (Figure 5A). B2R mRNA showed a biphasic pattern with an initial increase at day 2 (~12‐fold) and a second, higher peak at day 28 (~35‐fold) (Figure 5D). CD206 and YM1 mRNA levels peaked at day 2 (~2.2‐fold and ~4.5‐fold, respectively), followed by a sharp downregulation below baseline by Day 7, which persisted through Day 28 (Figure 5B,C). IL‐6 mRNA expression showed peaks at Day 2 (~200‐fold) and Day 28 (~350‐fold), with low levels between (Figure 5E). TNF‐*α* mRNA peaked at Day 2 (~3.2‐fold) and remained moderately elevated through Day 28 (Figure 5F).

## 4 Discussion

This study explores the roles of both kinin receptors in the acute and chronic phases of folic acid‐induced nephropathy, with a focus on the involvement of M1 and M2 macrophage polarization. Previously, we demonstrated that B1R antagonism and deletion had a protective effect in the transition from AKI to CKD in an ischemia–reperfusion model [23]. In the present study, we further investigate the roles of both kinin B1R and B2R in this context.

Since the kallikrein–kinin system (KKS) plays a pivotal role in the initiation and propagation of inflammatory responses, acute activation of the system leads to the release of kinins, such as bradykinin, which increase vascular permeability, induce vasodilation and mediate nociception primarily through activation of the constitutively expressed B₂ receptor. In addition to these direct vascular effects, kinins stimulate the production of secondary inflammatory mediators, including nitric oxide (NO) and prostacyclin, which further modulate vascular tone and facilitate the development of edema and leukocyte recruitment. During chronic inflammation, persistent activation of the system upregulates the inducible B₁ receptor and interacts with proinflammatory cytokines, thereby sustaining pain, promoting tissue remodelling, and contributing to the pathogenesis of chronic inflammatory diseases such as rheumatoid arthritis. Moreover, kinin signalling enhances prostaglandin synthesis and activates phospholipase‐dependent pathways, amplifying downstream inflammatory cascades. Collectively, these mechanisms underscore the dual role of the KKS in coordinating both the acute onset and chronic maintenance of inflammatory processes through integrated vascular and mediator‐driven effects [15, 18, 33].

We utilized the folic acid‐induced nephropathy model, as it is a well‐established model that effectively balances simplicity and relevance to human kidney diseases and is widely used in preclinical drug development [34]. In this study, we evaluated the roles of B1R and B2R antagonism during both the acute phase (48‐h postfolic acid injection) and the chronic phase (28‐day postfolic acid injection).

Kinin B1R antagonism has previously shown efficacy in AKI models of ischemia–reperfusion and cisplatin nephrotoxicity, where it reduced creatinine and urea levels, lowered tubular injury scores, and modulated the mRNA expression of proinflammatory cytokines [20, 23]. Interestingly, in the present study, B1R antagonism did not prevent AKI; rather, it exacerbated the condition, leading to increased creatinine and urea levels, as well as an elevated tubular injury score. Conversely, we have previously observed that kinin B2R antagonism attenuates cisplatin‐induced nephrotoxicity [21]. However, in the present study, kinin B2R antagonism failed to alleviate folic acid‐induced renal dysfunction. Interestingly, it was still able to mitigate tubular injury; these discrepancies underscore the differential mechanisms of injury in AKI animal models from different aetiologies and the importance of evaluating the efficacy of novel therapeutic agents to assess their generalizability.

One of the key components in the inflammatory response is macrophages, which exist in two subtypes with distinct functions. M1 macrophages are primarily involved in proinflammatory processes, while M2 macrophages play a crucial role in tissue repair and resolution of inflammation [35, 36]. In general, in injured kidneys, an increased presence of M1 macrophages correlates with increased injury severity characterized by elevated blood urea levels and more extensive tubular injury, indicating greater tissue damage and progressive development of kidney fibrosis. In contrast, an increased presence of M2 macrophages in the acute phase after injury has been associated with reduced kidney damage and a better recovery following an acute insult, leading to less kidney fibrosis [9, 37, 38]. In our study, we observed that B1R antagonism treatment did not reduce mRNA levels of M1 macrophages but was able to increase the expression of M2 macrophage markers, as previously observed in our earlier study using the renal ischemia–reperfusion model [23]. In contrast, treatment with the kinin B2R antagonist was able to reduce the levels of IL‐1*β*, a proinflammatory cytokine and increase the expression of the M2 macrophage marker CD206. B2R is expressed in monocytes and macrophages, key cells involved in IL‐1*β* production during the acute inflammatory response. Activation of B2R in these cells strongly promotes the synthesis and secretion of IL‐1*β*, more prominently than other cytokines such as IL‐6 or TNF‐*α*, which may also be regulated by additional pathways independent of B2R [19, 39]. The role of KKS in the inflammatory process has been well established. The B1R agonist can induce the release of inflammatory mediators such as IL‐1*β* and TNF, while B1RKO mice are protected from the effects of LPS on blood pressure. On the other hand, B1R also has a direct effect on neutrophils, promoting their migration to sites of inflammation [17]. The B2 receptor (B2R) plays a crucial role in inflammation. Inflamed tissues release bradykinin, which binds to B2R, perpetuating inflammation and tissue damage [40].

AKI can progress to CKD through a process characterized by inflammation, maladaptive repair and fibrosis. Infiltrating inflammatory cells play a crucial role in both the injury and repair phases following an acute insult. During the early phase, macrophages promote inflammation and exacerbate tissue injury. In the later phase, macrophages are essential for the repair process, functioning as scavengers of cellular debris and facilitating tissue regeneration [41, 42]. In this study, we observed that treatment with the kinin B1R antagonist was able to mitigate folic acid‐induced CKD, an effect that was similarly observed with kinin B2R antagonist treatment.

It is well established that kinin B2R mediates more acute responses [43]. Indeed, we observed this through a time‐course analysis of B2R mRNA levels, which showed kinetics that overlap with those of several proinflammatory cytokines (Figure 5). This suggests that B2R antagonism may acutely modulate M1 macrophage markers, thereby preventing the progression of CKD.

In contrast, kinin B1R is well known to be associated with the chronic phase of tissue injury [33, 43], as demonstrated by the time‐course analysis of B1R mRNA expression. The kinetics of B1R expression did not correlate with the markers of either M1 or M2 macrophages. However, we observed that as B1R mRNA expression begins to peak, the expression of M2 macrophage markers decreases, suggesting a potential relationship (Figure 5). Notably, treatment with the kinin B1R antagonist resulted in an increase in M2 macrophage markers, which may represent an important mechanism by which this treatment mitigates the progression of CKD and development of fibrosis. Following the acute insult, as the clearance process progresses, proinflammatory macrophages (M1 phenotype) progressively polarize into anti‐inflammatory macrophages (M2 phenotype), which mitigate the tissue damage caused by M1 macrophages and promote wound healing and tissue repair [44].

## 5 Conclusion

In conclusion, this study demonstrates that kinin receptors play a role in preventing folic acid‐induced fibrosis. Specifically, kinin B1R antagonism appears to modulate M2 macrophage markers, while kinin B2R antagonism is closely associated with the modulation of M1 macrophage markers. Further studies are required to elucidate the underlying mechanisms; however, these findings provide a valuable direction for future research aimed at identifying effective therapeutic strategies to attenuate the development of tubular interstitial fibrosis.

## Funding

This research was funded by grants from the Fundação de Amparo a Pesquisa do Estado de São Paulo (FAPESP grant 2020/15895‐7, 2024/22400‐5 and 2021/14313‐7).

## Conflicts of Interest

The authors declare no conflicts of interest.

## Data Availability

All data generated for this study are in this article.

## References

[1] P. Romagnani , R. Agarwal , J. C. N. Chan , et al., “Chronic Kidney Disease,” Nature Reviews. Disease Primers 11 (2025): 8. 10.1038/s41572-024-00589-9 39885176

[2] A. S. Levey , K. U. Eckardt , Y. Tsukamoto , et al., “Definition and Classification of Chronic Kidney Disease: A Position Statement From Kidney Disease: Improving Global Outcomes (KDIGO),” Kidney International 67 (2005): 2089–2100. 15882252 10.1111/j.1523-1755.2005.00365.x

[3] A. S. Levey , R. Atkins , J. Coresh , et al., “Chronic Kidney Disease as a Global Public Health Problem: Approaches And Initiatives—A Position Statement From Kidney Disease Improving Global Outcomes,” Kidney International 72 (2007): 247–259. 17568785 10.1038/sj.ki.5002343

[4] H. N. Johnson and L. Prasad‐Reddy , “Updates in Chronic Kidney Disease,” Journal of Pharmacy Practice 37 (2024): 1380–1390. 38877746 10.1177/08971900241262381

[5] A. Agarwal , X. Zeng , S. Li , et al., “Sodium‐Glucose Cotransporter‐2 (SGLT‐2) Inhibitors for Adults With Chronic Kidney Disease: A Clinical Practice Guideline,” BMJ 387 (2024): e080257. 39353639 10.1136/bmj-2024-080257

[6] B. M. Klinkhammer and P. Boor , “Kidney Fibrosis: Emerging Diagnostic and Therapeutic Strategies,” Molecular Aspects of Medicine 93 (2023): 101206. 37541106 10.1016/j.mam.2023.101206

[7] J. Barrera‐Chimal , S. Girerd , and F. Jaisser , “Mineralocorticoid Receptor Antagonists and Kidney Diseases: Pathophysiological Basis,” Kidney International 96 (2019): 302–319. 31133455 10.1016/j.kint.2019.02.030

[8] R. Huang , P. Fu , and L. Ma , “Kidney Fibrosis: From Mechanisms to Therapeutic Medicines,” Signal Transduction and Targeted Therapy 8 (2023): 129. 36932062 10.1038/s41392-023-01379-7 PMC10023808

[9] J. Barrera‐Chimal , G. R. Estrela , S. M. Lechner , et al., “The Myeloid Mineralocorticoid Receptor Controls Inflammatory and Fibrotic Responses After Renal Injury via Macrophage Interleukin‐4 Receptor Signaling,” Kidney International 93 (2018): 1344–1355. 29548765 10.1016/j.kint.2017.12.016

[10] J. Zeng , Y. Zhang , and C. Huang , “Macrophages Polarization in Renal Inflammation and Fibrosis Animal Models (Review),” Molecular Medicine Reports 29 (2024): 1–17. 38131228 10.3892/mmr.2023.13152 PMC10784723

[11] P. G. McLean , A. Ahluwalia , and M. Perretti , “Association Between Kinin B(1) Receptor Expression and Leukocyte Trafficking Across Mouse Mesenteric Postcapillary Venules,” Journal of Experimental Medicine 192 (2000): 367–380. 10934225 10.1084/jem.192.3.367 PMC2193221

[12] J. B. Pesquero , R. C. Araujo , P. A. Heppenstall , et al., “Hypoalgesia and Altered Inflammatory Responses in Mice Lacking Kinin B1 Receptors,” Proceedings of the National Academy of Sciences of the United States of America 97 (2000): 8140–8145. 10859349 10.1073/pnas.120035997 PMC16683

[13] S. Koyama , E. Sato , H. Numanami , K. Kubo , S. Nagai , and T. Izumi , “Bradykinin Stimulates Lung Fibroblasts to Release Neutrophil and Monocyte Chemotactic Activity,” American Journal of Respiratory Cell and Molecular Biology 22 (2000): 75–84. 10615068 10.1165/ajrcmb.22.1.3752

[14] E. Sato , S. Koyama , H. Nomura , K. Kubo , and M. Sekiguchi , “Bradykinin Stimulates Alveolar Macrophages to Release Neutrophil, Monocyte, and Eosinophil Chemotactic Activity,” Journal of Immunology 157 (1996): 3122–3129. 8816423

[15] J. Duchene and A. Ahluwalia , “The Kinin B(1) Receptor and Inflammation: New Therapeutic Target for Cardiovascular Disease,” Current Opinion in Pharmacology 9 (2009): 125–131. 19124274 10.1016/j.coph.2008.11.011

[16] A. B. Brechter , E. Persson , I. Lundgren , and U. H. Lerner , “Kinin B1 and B2 Receptor Expression in Osteoblasts and Fibroblasts Is Enhanced by Interleukin‐1 and Tumour Necrosis Factor‐Alpha. Effects Dependent on Activation of NF‐kappaB and MAP Kinases,” Bone 43 (2008): 72–83. 18467203 10.1016/j.bone.2008.02.003

[17] R. C. Araújo , R. Kettritz , I. Fichtner , A. C. Paiva , J. B. Pesquero , and M. Bader , “Altered Neutrophil Homeostasis in Kinin B1 Receptor‐Deficient Mice,” Biological Chemistry 382 (2001): 91–95. 11258678 10.1515/BC.2001.014

[18] M. E. Moreau , N. Garbacki , G. Molinaro , N. J. Brown , F. Marceau , and A. Adam , “The Kallikrein‐Kinin System: Current and Future Pharmacological Targets,” Journal of Pharmacological Sciences 99 (2005): 6–38. 16177542 10.1254/jphs.srj05001x

[19] F. Qadri and M. Bader , “Kinin B1 Receptors as a Therapeutic Target for Inflammation,” Expert Opinion on Therapeutic Targets 22 (2018): 31–44. 29168929 10.1080/14728222.2018.1409724

[20] G. R. Estrela , F. Wasinski , D. C. Almeida , et al., “Kinin B1 Receptor Deficiency Attenuates Cisplatin‐Induced Acute Kidney Injury by Modulating Immune Cell Migration,” Journal of Molecular Medicine (Berlin, Germany) 92 (2014): 399–409. 24357263 10.1007/s00109-013-1116-z

[21] G. R. Estrela , F. Wasinski , R. F. Bacurau , D. M. Malheiros , N. O. Camara , and R. C. Araujo , “Kinin B2 Receptor Deletion and Blockage Ameliorates Cisplatin‐Induced Acute Renal Injury,” International Immunopharmacology 22 (2014): 115–119. 24975837 10.1016/j.intimp.2014.06.025

[22] D. Basuli , R. U. Parekh , A. White , A. Thayyil , and S. Sriramula , “Kinin B1 Receptor Mediates Renal Injury and Remodeling in Hypertension,” Frontiers Medecine (Lausanne) 8 (2021): 780834. 10.3389/fmed.2021.780834 PMC8804098 35118089

[23] G. R. Estrela , R. B. Santos , A. Budu , A. C. de Arruda , J. Barrera‐Chimal , and R. C. Araújo , “Kinin B1 Receptor Antagonism Prevents Acute Kidney Injury to Chronic Kidney Disease Transition in Renal Ischemia‐Reperfusion by Increasing the M2 Macrophages Population in C57BL6J Mice,” Biomedicine 11 (2023): 2194. 10.3390/biomedicines11082194 PMC10452634 37626691

[24] J. Klein , J. Gonzalez , J. Duchene , et al., “Delayed Blockade of the Kinin B1 Receptor Reduces Renal Inflammation and Fibrosis in Obstructive Nephropathy,” FASEB Journal 23 (2009): 134–142. 18809736 10.1096/fj.08-115600

[25] J. Klein , J. Gonzalez , S. Decramer , et al., “Blockade of the Kinin B1 Receptor Ameloriates Glomerulonephritis,” Journal of the American Society of Nephrologyl 21 (2010): 1157–1164. 10.1681/ASN.2009090887 PMC3152233 20448019

[26] W. C. Chiang , C. T. Chien , W. W. Lin , et al., “Early Activation of Bradykinin B2 Receptor Aggravates Reactive Oxygen Species Generation and Renal Damage in Ischemia/Reperfusion Injury,” Free Radical Biology & Medicine 41 (2006): 1304–1314. 17015177 10.1016/j.freeradbiomed.2006.07.011

[27] A. Budu , L. C. Freitas‐Lima , A. C. Arruda , et al., “Renal Fibrosis due to Multiple Cisplatin Treatment Is Exacerbated by Kinin B1 Receptor Antagonism,” Brazilian Journal of Medical and Biological Research 54 (2021): e11353. 34669782 10.1590/1414-431X2021e11353 PMC8521536

[28] G. R. Estrela , L. C. Freitas‐Lima , A. Budu , et al., “Chronic Kidney Disease Induced by Cisplatin, Folic Acid and Renal Ischemia Reperfusion Induces Anemia and Promotes GATA‐2 Activation in Mice,” Biomedicine 9 (2021): 769. 10.3390/biomedicines9070769 PMC8301442 34356833

[29] L. J. Yan , “Folic Acid‐Induced Animal Model of Kidney Disease,” Animal Models and Experimental Medicine 4 (2021): 329–342. 34977484 10.1002/ame2.12194 PMC8690981

[30] P. Tveden‐Nyborg , T. K. Bergmann , N. Jessen , U. Simonsen , and J. Lykkesfeldt , “BCPT 2023 Policy for Experimental and Clinical Studies,” Basic & Clinical Pharmacology & Toxicology 133 (2023): 391–396. 37732406 10.1111/bcpt.13944

[31] G. R. Estrela , F. Wasinski , R. J. F. Felizardo , et al., “MATE‐1 Modulation by Kinin B1 Receptor Enhances Cisplatin Efflux From Renal Cells,” Molecular and Cellular Biochemistry 428 (2017): 101–108. 28161805 10.1007/s11010-016-2920-x

[32] A. F. Viana , I. S. Maciel , F. N. Dornelles , et al., “Kinin B1 Receptors Mediate Depression‐Like Behavior Response in Stressed Mice Treated With Systemic *E. coli* Lipopolysaccharide,” Journal of Neuroinflammation 7 (2010): 98. 21194425 10.1186/1742-2094-7-98 PMC3022820

[33] J. B. Calixto , R. Medeiros , E. S. Fernandes , J. Ferreira , D. A. Cabrini , and M. M. Campos , “Kinin B1 Receptors: Key G‐Protein‐Coupled Receptors and Their Role in Inflammatory and Painful Processes,” British Journal of Pharmacology 143 (2004): 803–818. 15520046 10.1038/sj.bjp.0706012 PMC1575942

[34] J. D. Morel , M. B. Sleiman , T. Y. Li , et al., “Mitochondrial and NAD+ Metabolism Predict Recovery From Acute Kidney Injury in a Diverse Mouse Population,” JCI Insight 8 (2023): e164626. 36752209 10.1172/jci.insight.164626 PMC9977436

[35] S. Chen , A. F. U. H. Saeed , Q. Liu , et al., “Macrophages in Immunoregulation and Therapeutics,” Signal Transduction and Targeted Therapy 8 (2023): 207. 37211559 10.1038/s41392-023-01452-1 PMC10200802

[36] Z. Strizova , I. Benesova , R. Bartolini , et al., “M1/M2 Macrophages and Their Overlaps—Myth or Reality?,” Clinical Science (London, England) 137 (2023): 1067–1093. 10.1042/CS20220531 PMC10407193 37530555

[37] S. Tian and S. Y. Chen , “Macrophage Polarization in Kidney Diseases,” Macrophage (Houst) 2 (2015): e679. 26082946 10.14800/macrophage.679 PMC4465799

[38] H. I. Han , L. B. Skvarca , E. B. Espiritu , A. J. Davidson , and N. A. Hukriede , “The Role of Macrophages During Acute Kidney Injury: Destruction and Repair,” Pediatric Nephrology 34 (2019): 561–569. 29383444 10.1007/s00467-017-3883-1 PMC6066473

[39] H. Shirasaki , E. Kanaizumi , and T. Himi , “Immunohistochemical Localization of the Bradykinin B1 and B2 Receptors in Human Nasal Mucosa,” Mediators of Inflammation 2009 (2009): 102406. 19404481 10.1155/2009/102406 PMC2673475

[40] D. A. B. Rex , K. Deepak , N. Vaid , et al., “A Modular Map of Bradykinin‐Mediated Inflammatory Signaling Network,” Journal of Cell Communication and Signaling 16 (2022): 301–310. 34714516 10.1007/s12079-021-00652-0 PMC8554507

[41] D. P. Basile , J. V. Bonventre , R. Mehta , et al., “Progression After AKI: Understanding Maladaptive Repair Processes to Predict and Identify Therapeutic Treatments,” Journal of the American Society of Nephrologyl 27 (2016): 687–697. 10.1681/ASN.2015030309 PMC4769207 26519085

[42] T. Zhang , R. E. Widdop , and S. D. Ricardo , “Transition From Acute Kidney Injury to Chronic Kidney Disease: Mechanisms, Models, and Biomarkers,” American Journal of Physiology. Renal Physiology 327 (2024): F788–F805. 39298548 10.1152/ajprenal.00184.2024

[43] D. G. Souza , E. S. Lomez , V. Pinho , et al., “Role of Bradykinin B2 and B1 Receptors in the Local, Remote, and Systemic Inflammatory Responses That Follow Intestinal Ischemia and Reperfusion Injury,” Journal of Immunology 172 (2004): 2542–2548. 10.4049/jimmunol.172.4.2542 14764727

[44] G. Li , H. Yang , D. Zhang , et al., “The Role of Macrophages in Fibrosis of Chronic Kidney Disease,” Biomedicine & Pharmacotherapy 177 (2024): 117079. 38968801 10.1016/j.biopha.2024.117079

